# Improved Estimation and Interpretation of Correlations in Neural Circuits

**DOI:** 10.1371/journal.pcbi.1004083

**Published:** 2015-03-31

**Authors:** Dimitri Yatsenko, Krešimir Josić, Alexander S. Ecker, Emmanouil Froudarakis, R. James Cotton, Andreas S. Tolias

**Affiliations:** 1 Department of Neuroscience, Baylor College of Medicine, Houston, Texas, United States of America; 2 Department of Mathematics and Department of Biology and Biochemistry, University of Houston, Houston, Texas, United States of America; 3 Werner Reichardt Center for Integrative Neuroscience and Institute for Theoretical Physics, University of Tübingen, Germany; 4 Bernstein Center for Computational Neuroscience, Tübingen, Germany; 5 Max Planck Institute for Biological Cybernetics, Tübingen, Germany; 6 Department of Computational and Applied Mathematics, Rice University, Houston, Texas, United States of America; The University of Texas at Austin, UNITED STATES

## Abstract

Ambitious projects aim to record the activity of ever larger and denser neuronal populations in vivo. Correlations in neural activity measured in such recordings can reveal important aspects of neural circuit organization. However, estimating and interpreting large correlation matrices is statistically challenging. Estimation can be improved by regularization, i.e. by imposing a structure on the estimate. The amount of improvement depends on how closely the assumed structure represents dependencies in the data. Therefore, the selection of the most efficient correlation matrix estimator for a given neural circuit must be determined empirically. Importantly, the identity and structure of the most efficient estimator informs about the types of dominant dependencies governing the system. We sought statistically efficient estimators of neural correlation matrices in recordings from large, dense groups of cortical neurons. Using fast 3D random-access laser scanning microscopy of calcium signals, we recorded the activity of nearly every neuron in volumes 200 μm wide and 100 μm deep (150–350 cells) in mouse visual cortex. We hypothesized that in these densely sampled recordings, the correlation matrix should be best modeled as the combination of a sparse graph of pairwise partial correlations representing local interactions and a low-rank component representing common fluctuations and external inputs. Indeed, in cross-validation tests, the covariance matrix estimator with this structure consistently outperformed other regularized estimators. The sparse component of the estimate defined a graph of interactions. These interactions reflected the physical distances and orientation tuning properties of cells: The density of positive ‘excitatory’ interactions decreased rapidly with geometric distances and with differences in orientation preference whereas negative ‘inhibitory’ interactions were less selective. Because of its superior performance, this ‘sparse+latent’ estimator likely provides a more physiologically relevant representation of the functional connectivity in densely sampled recordings than the sample correlation matrix.

## Author Summary

It is now possible to record the spiking activity of hundreds of neurons at the same time. A meaningful statistical description of the collective activity of these neural populations—their ‘functional connectivity’—is a forefront challenge in neuroscience. We addressed this problem by identifying statistically efficient estimators of correlation matrices of the spiking activity of neural populations. Various underlying processes may reflect differently on the structure of the correlation matrix: Correlations due to common network fluctuations or external inputs are well estimated by low-rank representations, whereas correlations arising from linear interactions between pairs of neurons are well approximated by their pairwise *partial* correlations. In our data obtained from fast 3D two-photon imaging of calcium signals of large and dense groups of neurons in mouse visual cortex, the best estimation performance was attained by decomposing the correlation matrix into a sparse network of partial correlations (‘interactions’) combined with a low-rank component. The inferred interactions were both positive (‘excitatory’) and negative (‘inhibitory’) and reflected the spatial organization and orientation preferences of the interacting cells. We propose that the most efficient among many estimators provides a more informative picture of the functional connectivity than previous analyses of neural correlations.

## Introduction

*Functional connectivity* is a statistical description of observed *multineuronal* activity patterns not reducible to the response properties of the individual cells. Functional connectivity reflects local synaptic connections, shared inputs from other regions, and endogenous network activity. Although functional connectivity is a phenomenological description without a strict mechanistic interpretation, it can be used to generate hypotheses about the anatomical architecture of the neural circuit and to test hypotheses about the processing of information at the population level.

Pearson correlations between the spiking activity of pairs of neurons are among the most familiar measures of functional connectivity [1–5]. In particular, *noise correlations*, *i.e.* the correlations of trial-to-trial response variability between pairs of neurons, have a profound impact on stimulus coding [1, 2, 6–11]. In addition, noise correlations and correlations in spontaneous activity have been hypothesized to reflect aspects of synaptic connectivity [12]. Interest in neural correlations has been sustained by a series of discoveries of their nontrivial relationships to various aspects of circuit organization such as the physical distances between the neurons [13, 14], their synaptic connectivity [15], stimulus response similarity [3–5, 15–22], cell types [23], cortical layer specificity [24, 25], progressive changes in development and in learning [26–28], changes due to sensory stimulation and global brain states [21, 29–33].

Neural correlations do not come with ready or unambiguous mechanistic interpretations. They can arise from monosynaptic or polysynaptic interactions, common or correlated inputs, oscillations, top-down modulation, and background network fluctuations, and other mechanisms [34–39]. But multineuronal recordings do provide more information than an equivalent number of separately recorded pairs of cells. For example, the eigenvalue decomposition of the covariance matrix expresses shared correlated activity components across the population; common fluctuations of population activity may be accurately represented by only a few eigenvectors that affect all correlation coefficients. On the other hand, a correlation matrix can be specified using the *partial correlations* between pairs of the recorded neurons. The partial correlation coefficient between two neurons reflects their linear association conditioned on the activity of all the other recorded cells [40]. Under some assumptions, partial correlations measure conditional independence between variables and may more directly approximate causal effects between components of complex systems than correlations [40]. For this reason, partial correlations have been used to describe interactions between genes in functional genomics [41, 42] and between brain regions in imaging studies [43, 44]. These opportunities have not yet been explored in neurophysiological studies where most analyses have only considered the distributions of pairwise correlations [2, 4, 5, 13].

However, estimation of correlation matrices from large populations presents a number of numerical challenges. The amount of recorded data grows only linearly with population size whereas the number of estimated coefficients increases quadratically. This mismatch leads to an increase in spurious correlations, overestimation of common activity (*i.e.* overestimation of the largest eigenvalues) [45], and poorly conditioned partial correlations [41]. The *sample correlation matrix* is an unbiased estimate of the true correlations but its many free parameters make it sensitive to sampling noise. As a result, on average, the sample correlation matrix is farther from the true correlation matrix than some structured estimates.

Estimation can be improved through *regularization*, the technique of deliberately imposing a structure on an estimate in order to reduce its estimation error [41, 46]. To ‘impose a structure’ on an estimate means to bias (‘shrink’) it toward a reduced representation with fewer free parameters, the *target estimate*. The optimal target estimate and the optimal amount of shrinkage can be obtained from the data sample either analytically [41, 45, 47] or by cross-validation [48]. An estimator that produces estimates that are, on average, closer to the truth for a given sample size is said to be more *efficient* than other estimators.

Although regularized covariance matrix estimation is commonplace in finance [47], functional genomics [41], and brain imaging [44], surprisingly little work has been done to identify optimal regularization of neural correlation matrices.

Improved estimation of the correlation matrix is beneficial in itself. For example, improved estimates can be used to optimize decoding of the population activity [48, 49]. But reduced estimation error is not the only benefit of regularization. Finding the most efficient among many regularized estimators leads to insights about the system itself: the structure of the most efficient estimator is a parsimonious representation of the regularities in the data.

The advantages due to regularization increase with the size of the recorded population. With the advent of big neural data [50], the search for optimal regularization schemes will become increasingly relevant in any model of population activity. Since optimal regularization schemes are specific to systems under investigation, the inference of functional connectivity in large-scale neural data will entail the search for optimal regularization schemes. Such schemes may involve combinations of heuristic rules and numerical techniques specially designed for given types of neural circuits.

What structures of correlation matrices best describe the multineuronal activity in specific circuits and in specific brain states? More specifically, are correlations in the visual cortex during visual stimulation best explained by common fluctuations or by local interactions within the recorded microcircuit?

To address these questions, we evaluated four regularized covariance matrix estimators that imposed different structures on the estimate. The estimators are designated as follows:

*C*_sample_—the sample covariance matrix, the unbiased estimator.
*C*_diag_—linear shrinkage of covariances toward zero, *i.e.* toward a diagonal covariance matrix.
*C*_factor_—a low-rank approximation of the sample covariance matrix, representing inputs from unobserved shared factors (latent units).
*C*_sparse_—sparse partial correlations, *i.e.* a large fraction of the *partial* correlations between pairs of neurons are set to zero.
*C*_sparse+latent_—sparse partial correlations between the recorded neurons *and* linear interactions with a number of latent units.

First, we used simulated data to demonstrate that the selection of the optimal estimator indeed pointed to the true structure of the dependencies in the data.

We then performed a cross-validated evaluation to establish which of the four regularized estimators was most efficient for representing the population activity of dense groups of neurons in mouse primary visual cortex recorded with high-speed 3D random-access two-photon imaging of calcium signals. In our data, the sample correlation coefficients were largely positive and low. We found that the most efficient estimator of the correlation matrix in these data was *C*_sparse+latent_. This estimator revealed a sparse network of partial correlations (‘interactions’), between the observed neurons; it also inferred a number of latent units interacting with the observed neurons. We analyzed these networks of partial correlations and found the following: Whereas significant noise correlations were predominantly positive, the inferred interactions had a large fraction of negative values possibly reflecting inhibitory circuitry. Moreover, the inferred positive interactions exhibited a substantially stronger relationship to the physical distances and to the differences in preferred orientations than noise correlations. In contrast, the inferred negative interactions were less selective.

## Results

### Covariance estimation

The covariance matrix is defined as

$$\Sigma =\;E\left[(x-\mu ){\left(x-\mu \right)}^{T}\right],\;\mu =\;E\left[x\right]$$ (1)

where the *p* × 1 vector *x* is a single observation of the firing rates of *p* neurons in a time bin of some duration, E[⋅] denotes expectation, and *μ* is the vector of expected firing rates.

Given a set of observations {*x*(*t*): *t* ∈ *T*} of population activity, where *x*(*t*) contains observed firing rates in time bin *t*, and an independent estimate of the mean firing rates $\bar{x}$, the *sample covariance matrix*,

$$C_{\text{sample}}=\frac{1}{n}\sum_{t\in T}(x(t)-\bar{x}){\left(x(t)-\bar{x}\right)}^{T},$$ (2)

where *n* is the number of time bins in *T*, is an unbiased estimate of the true covariance matrix, *i.e.* $E\left[C_{\text{sample}}\right]=\Sigma$. In all cases when the unbiasedness of the sample covariance matrix matters in this paper, the mean activity is estimated independently from a separate sample.

Given any covariance matrix estimate *C*, the corresponding correlation matrix *R* is calculated by normalizing the rows and columns of *C* by the square roots of its diagonal elements to produce unit entries on the diagonal:

$$R={\left(\text{diag}(C)\right)}^{-\frac{1}{2}}C{\left(\text{diag}(C)\right)}^{-\frac{1}{2}},$$ (3)

where diag(*C*) denotes the diagonal matrix with the diagonal elements from *C*.

The *partial correlation* between a pair of variables is the Pearson correlation coefficient of the residuals of the linear least-squares predictor of their activity based on all the other variables, excluding the pair [40, 51]. Partial correlations figure prominently in probabilistic *graphical modeling* wherein the joint distribution is explained by sets of pairwise interactions [40]. For multivariate Gaussian distributions, zero partial correlations indicate conditional independence of the pair, implying a lack of direct interaction [40, 52]. More generally, partial correlations can serve as a measure of conditional independence under the assumption that dependencies in the system are close to linear effects [40, 53]. As neural recordings become increasingly dense, partial correlations may prove useful as indicators of conditional independence (lack of functional connectivity) between pairs of neurons.

Pairwise partial correlations are closely related to the elements of the *precision matrix*, *i.e.* the inverse of the covariance matrix [40, 52]. Zero elements in the precision matrix signify zero partial correlations between the corresponding pairs of variables. Given the covariance estimate *C*, the matrix of partial correlations *P* is computed by normalizing the rows and columns of the *precision matrix* *C* ^−1^ to produce negative unit entries on the diagonal:

$$P=-{\left(\text{diag}(C^{-1})\right)}^{-\frac{1}{2}}\;C^{-1}{\left(\text{diag}(C^{-1})\right)}^{-\frac{1}{2}}$$ (4)

Increasing the number of recorded neurons results in a higher *condition number* of the sample covariance matrix [45] making the partial correlation estimates more *ill-conditioned*: small errors in the covariance estimates translate into greater errors in the estimates of the partial correlations. With massively multineuronal recordings, partial correlations cannot be estimated without *regularization* [41, 45].

**Fig 1:**
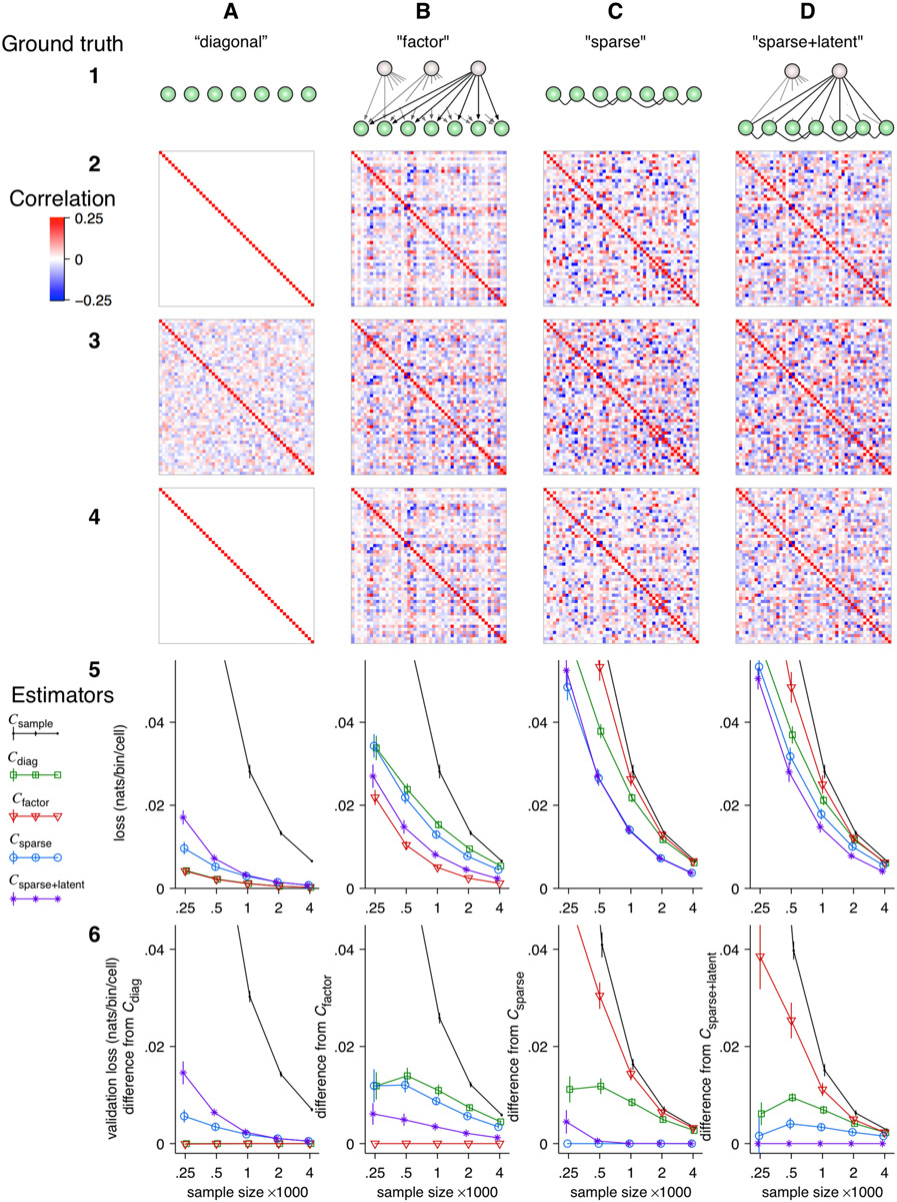
Regularized estimators whose structure matches the true structure in the data are more efficient. **Row 1.** Graphical models of the target estimates of the four respective regularized covariance matrix estimators. Recorded neurons are represented by the green spheres and latent units by the lightly shaded spheres. Edges represent conditional dependencies, *i.e.* ‘interactions’. **Row 1, A**. For estimator *C*_diag_, the target estimate is a diagonal matrix, which describes systems that lack linear dependencies. **Row 1, B.** For estimator *C*_factor_, the target estimate is a factor model (low-rank matrix plus a diagonal matrix), representing systems in which correlations arise due to common input from latent units. **Row 1, C**. For estimator *C*_sparse_, the covariance matrix is approximated as the inverse of a sparse matrix. This approximation describes systems in which correlations arise from a sparse set of linear associations between the observed units. **Row 1, D**. For estimator *C*_sparse+latent_, the covariance matrix is approximated as the inverse of the sum of a sparse matrix and a low-rank matrix. This approximation describes a model wherein correlations arise due to sparse associations between the recorded cells *and* due to several latent units. **Row 2:** Examples of 50 × 50 correlation matrices corresponding to each structure: **A.** the diagonal correlation matrix, **B.** a factor model with four latent units, **C.** a correlation matrix with 67% off-diagonal zeros in its inverse, and **D.** a correlation matrix whose inverse is the sum of a rank-3 matrix (*i.e.* three latent units) and a sparse matrix with 76% off-diagonal zeros. **Row 3:** Sample correlation matrices calculated from samples of size *n* = 500 drawn from simulated random processes with respective correlation matrices shown in Row 2. The structure of the sample correlation matrix is difficult to discern by eye. **Row 4:** Estimates computed from the same data as in Row 3 using structured estimators of the correct type, optimized by cross-validation. The regularized estimates are closer to the truth than the sample correlation matrices. **Row 5:** True loss (Eq. 9) for the five estimators as a function of sample size. The error bars indicate the standard deviation of the mean. Estimators with structure that matches the true model converged to zero faster than the other estimators. **Row 6:** Validation loss (Eq. 10) for the five estimators relative to the matching estimators for each type of ground truth. Error bars indicate the standard deviation of the mean. Differences in validation loss approximate differences in true loss.

We considered four regularized estimators based on distinct families of target estimates: *C*_diag_, *C*_factor_, *C*_sparse_, and *C*_sparse+latent_. In probabilistic models with exclusively linear dependencies, the target estimates of these estimators correspond to distinct families of graphical models (Fig. 1 Row 1).

The target estimate of estimator *C*_diag_ is the diagonal matrix *D* containing estimates of neurons’ variances. Regularization is achieved by linear *shrinkage* of the sample covariance matrix *C*_sample_ toward *D* as controlled by the scalar *shrinkage intensity* parameter *λ* ∈ [0, 1]:

$$C_{\text{diag}}=(1-\lambda )C_{\text{sample}}+\lambda D\;$$ (5)

The structure imposed by *C*_diag_ describes a population with no linear associations between the neurons (Fig. 1 Row 1, A). If sample correlations are largely spurious, *C*_diag_ is expected to be more efficient than other estimators.

Estimator *C*_factor_ approximates the covariance matrix by the factor model,

$$C_{\text{factor}}\;=\;L\;+\;D,$$ (6)

where *L* is a *p* × *p* symmetric positive semidefinite matrix with low rank and *D* is a diagonal matrix. This approximation is the basis for *factor analysis* [51], where matrix *L* represents covariances arising from latent factors. The rank of *L* corresponds to the number of latent factors. Matrix *D* contains the variances of the cells’ independent activity from the latent factors. The estimator is regularized by selecting the rank of *L* and by shrinking the independent variances in *D* toward their mean. The structure imposed by *C*_factor_ describes a population whose activity is linearly driven by a number of latent factors that affect many cells while direct interactions between the recorded cells are insignificant (Fig. 1 Row 1, B).

Estimator *C*_sparse_ is produced by approximating the sample covariance matrix by the inverse of a sparse matrix *S*:

$$C_{\text{sparse}}=S^{-1}.$$ (7)

The estimator is regularized by adjusting the sparsity (fraction of off-diagonal zeros) of *S*. The problem of finding the optimal set of non-zero elements in *S* is known as *covariance selection* [52]. The structure imposed by *C*_sparse_ describes conditions in which neural correlations arise from direct linear effects (‘interactions’) between some pairs of neurons (Fig. 1 Row 1, C).

Estimator *C*_sparse+latent_ is obtained by approximating the sample covariance matrix by a matrix whose inverse is the difference of a sparse component and a low-rank component:

$$C_{\text{sparse}+\text{latent}}={\left(S-L\right)}^{-1},$$ (8)

where *S* is a sparse matrix and *L* is a low-rank matrix. The estimator is regularized by adjusting the sparsity of *S* and the rank of *L*. See Methods for more detailed explanations. The structure imposed by *C*_sparse+latent_ favors conditions in which the activity of neurons is determined by linear effects between some observed pairs of neurons and linear effects from several latent units (Fig. 1 Row 1, D) [54, 55].

We refer to the sparse partial correlations in estimators *C*_sparse_ and *C*_sparse+latent_ as ‘interactions’.

### Simulation

We next demonstrated how the most efficient among different regularized estimators can reveal the structure of correlations. We constructed four families of 50 × 50 covariance matrices, each with structure that matched one of the four regularized estimators (Fig. 1 Row 2, A–D and Methods). We used these covariance matrices as the ground truth in multivariate Gaussian distributions with zero means and drew samples of various sizes. The sample correlation matrices from finite samples (*e.g.* *n* = 500 in Fig. 1 Row 3) were contaminated with sampling noise and their underlying structures were difficult to discern.

The evaluation of any covariance matrix estimator, *C*, is performed with respect to a *loss function ℓ*(*C*, Σ) to quantify its discrepancy from the truth, Σ. The loss function is chosen to attain its minimum when *C* = Σ. Here, in the role of the loss function we adopted the Kullback-Leibler divergence between multivariate normal distributions with equal means, scaled by $\frac{2}{p}$ to make its values comparable across different population sizes:

$$\ell (C,\Sigma )=\frac{2}{p}D_{KL}\left(N_{\Sigma }\|N_{C}\right)=\frac{1}{p}\left[\text{Tr}(C^{-1}\Sigma )+\ln\;\det\;C-\ln\;\det\;\Sigma -p\right]$$ (9)

Thus *ℓ*(*C*, *Σ*) is expressed in nats/neuron per time bin.

When the ground truth is not accessible, the loss cannot be computed directly but may be estimated from data through *validation*. In a validation procedure, a validation sample covariance matrix $C_{\text{sample}}^{\prime }$ is computed from a testing data set that is independent from the data used for computing *C*. Then the *validation loss* $ℒ(C,C_{\text{sample}}^{\prime })$ measures the discrepancy of *C* from $C_{\text{sample}}^{\prime }$. Here, in the role of validation loss, we adopted the negative multivariate normal log likelihood of *C* given $C_{\text{sample}}^{\prime }$, also scaled by $\frac{2}{p}$ and omitting the constant term:

$$L\left(C,C_{\text{sample}}^{\prime }\right)=\frac{1}{p}\left[\text{Tr}(C^{-1}C_{\text{sample}}^{\prime })+/\ln\;\det\;C\right]$$ (10)

Since L(⋅,⋅) is additive in its second argument and $C_{\text{sample}}^{\prime }$ is an unbiased estimate of Σ, then, for given *C* and Σ, the validation loss is an unbiased estimate of the true loss, up to a constant:

$$E\left[L\left(C,C_{\text{sample}}^{\prime }\right)\right]\;=\;L\left(C,E\left[C_{\text{sample}}^{\prime }\right]\right)=L\left(C,\Sigma \right)=\ell (C,\Sigma )+\text{const}.$$ (11)

Therefore, the validation procedure allows comparing the relative values of the loss attained by different covariance matrix estimators even without access to the ground truth.

We drew 30 independent samples with sample sizes *n* = 250, 500, 1000, 2000, and 4000 from each model and computed the loss *ℓ*(*C*, Σ) for each of the five estimators. The hyperparameters of the regularized estimators were optimized by nested cross-validation using only the data in the sample. All the regularized estimators produced better estimates (lower loss) than the sample covariance matrix. However, estimators whose structure matched the true model outperformed the other estimators (Fig. 1 Rows 4 and 5). The validation loss computed by ten-fold cross-validation (see Methods) accurately reproduced the relative values of the true loss as well as the rankings of the estimators even without access to the ground truth (Fig. 1 Row 6).

Note that when the ground truth had zero correlations (Column A), *C*_factor_ performed equally well to *C*_diag_ because it correctly inferred zero factors and only estimated the individual variances. Similarly, when the number of latent units was zero (Column C), *C*_sparse+latent_ performed nearly equally well to *C*_sparse_ because it correctly inferred zero latent units. With increasing sample sizes, all estimators converged to the ground truth (zero loss) but the estimators with correct structure outperformed the others even for large samples.

In Gaussian models, the pairwise partial correlations perfectly characterize the conditional dependencies between the variables. To demonstrate that estimator rankings were robust to deviations from Gaussian models, we repeated the same cross-validated evaluation using pairwise Ising models to generate the data. Ising models have been used to infer functional connectivity from neuronal spike trains [56]. Conveniently, the Ising model has equivalent mathematical form to the Gaussian distribution,

$$x∼\frac{1}{Z(J,h)}\exp\left(\frac{1}{2}x^{T}Jx+h^{T}x\right)$$ (12)

but the Ising model is defined on the multivariate binary domain rather than the continuous domain. Both models are maximum-entropy models constrained to match the mean firing rates and the covariance matrix [57]. The partition function *Z*(*J*, *h*) normalizes the distributions on the models’ respective domains. In the Gaussian model, the matrix −*J* ^−1^ is the covariance matrix; and the mean values are *μ* = *J* ^−1^ *h*. For the Ising model, *J* is the matrix of pairwise interactions and *h* is the vector of the cells’ individual activity drives, although they do not have a simple relationship to the means and the covariance matrix. Both distributions have the same structure of pairwise conditional dependencies: zeros in the matrix *J* indicate conditional independence between the corresponding pair of neurons.

**Fig 2:**
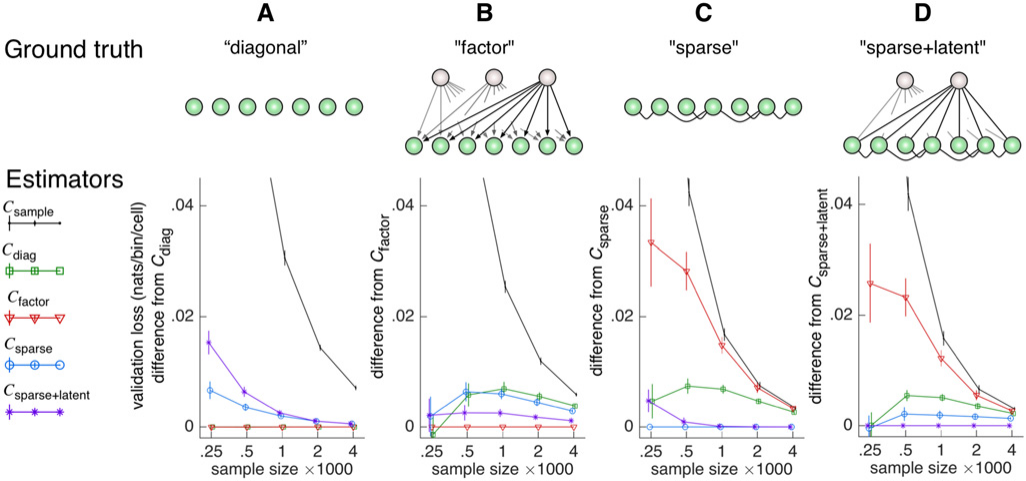
Performance of covariance estimators on samples drawn from Ising models. **A–D** Validation losses of covariance matrix estimators relative to the estimator whose structure matches the ground truth. The calculation is performed identically to Fig. 1 Row 6 except Ising models are used as ground truth.

Indeed, despite their considerable departure from strictly linear conditional dependencies, Ising models yielded the same relationships between the performances of the covariance estimators as the Gaussian models in cross-validation (Fig. 2). Identical interaction matrices *J* of the joint distributions over the observable and latent variables were used for both the Gaussian and the Ising models.

This simulation study demonstrated that cross-validated evaluation of regularized estimators of the covariance matrices of population activity can discriminate between structures of dependencies in the population. The selection of the most efficient covariance estimators for particular neural circuits is therefore an empirical finding characteristic of the nature of circuit interactions.

### The *C*_sparse+latent_ estimator is most efficient in neural data

**Fig 3:**
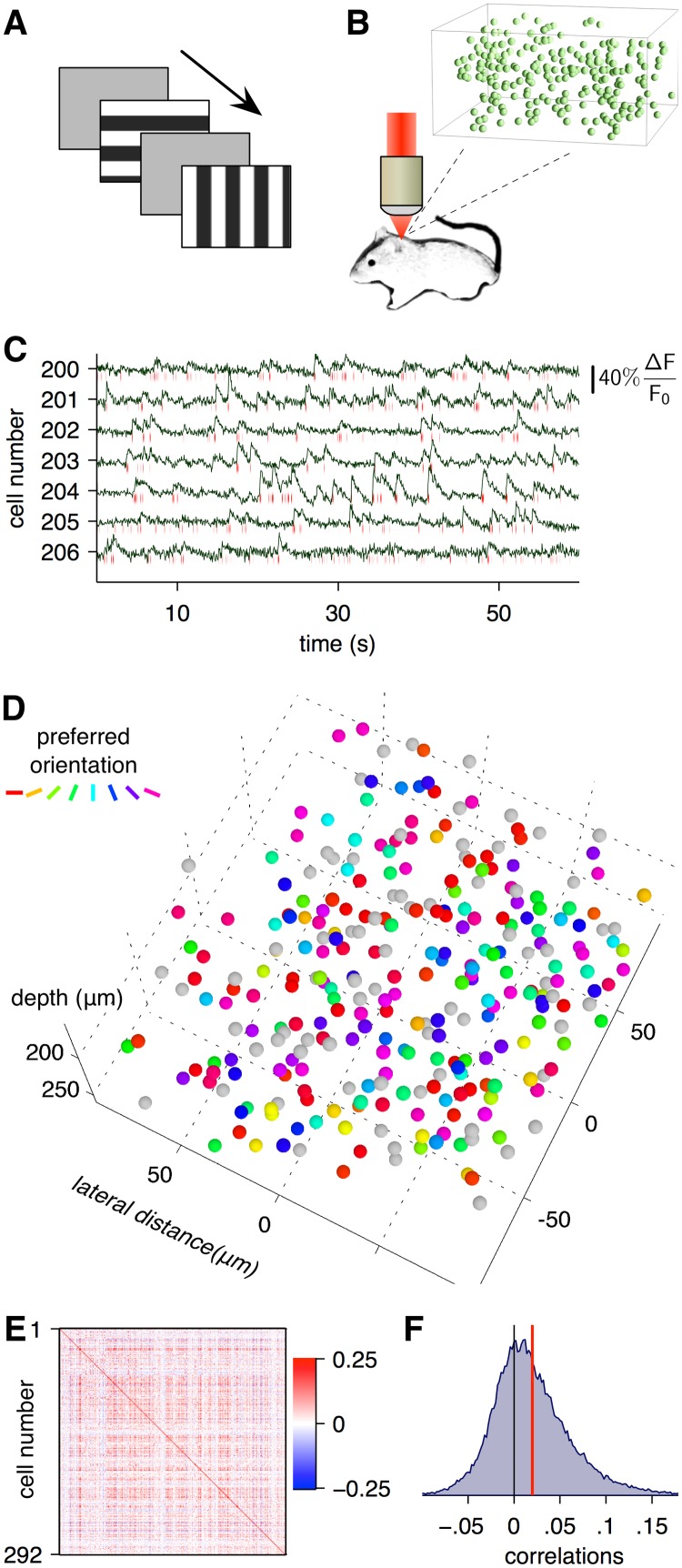
Acquisition of neural signals for the estimation of noise correlations. Visual stimuli comprising full-field drifting gratings interleaved with blank screens (**A**) presented during two-photon recordings of somatic calcium signals using fast 3D random-access microscopy (**B**). **C–F**. Calcium activity data from an example site. **C**. Representative calcium signals of seven cells, downsampled to 20 Hz, out of the 292 total recorded cells. Spiking activity inferred by nonnegative deconvolution is shown by red ticks below the trace. **D**. The spatial arrangement and orientation tuning of the 292 cells from the imaged site. The cells’ colors indicate their orientation preferences. The gray cells were not significantly tuned. **E**. The sample noise correlation matrix of the activity of the neural population. **F**. Histogram of noise correlation coefficients in one site. The red line indicates the mean correlation coefficient of 0.020.

We recorded the calcium activity of densely sampled populations of neurons in layers 2/3 and upper layer 4 in primary visual cortex of sedated mice using fast random-access 3D scanning two-photon microscopy during visual stimulation (Fig. 3 A–B) [58–60]. This technique allowed fast sampling (100–150 Hz) from large numbers (150–350) of cells in 200 × 200 × 100 *μ*m^3^ volumes of cortical tissue (Fig. 3 C and D). The instantaneous firing rates were inferred using sparse nonnegative deconvolution [61] (Fig. 3 C). Only cells that produced detectable calcium activity were included in the analysis (see Methods). First, 30 repetitions of full-field drifting gratings of 16 directions were presented in random order. Each grating was played for 500 ms, without intervening blanks. This stimulus was used to compute the orientation tuning of the recorded cells (Fig. 3 D). To estimate the noise correlation matrix, we presented only two distinct directions in some experiments or five directions in others with 100–300 repetitions of each condition. Each grating lasted 1 second and was followed by a 1-second blank. The traces were then binned into 150 ms intervals aligned on the stimulus onset for the estimation of the correlation matrix. The sample correlation coefficients were largely positive and low (Fig. 3 E and F). The average value of the correlation coefficient across sites ranged from 0.0065 to 0.051 with the mean across sites of 0.018.

In these densely sampled populations, direct interactions between cells are likely to influence the patterns of population activity. We therefore hypothesized that covariance matrix estimators that explicitly modeled the partial correlations between pairs of neurons (*C*_sparse_ and *C*_sparse+latent_) would have a performance advantage. However, the observed neurons must also be strongly influenced by global activity fluctuations and by unobserved common inputs to the advantage of estimators that explicitly model common fluctuations of the entire population: *C*_factor_ and *C*_sparse+latent_. If both types of effects are significant, then *C*_sparse+latent_ should outperform the other estimators.

**Fig 4:**
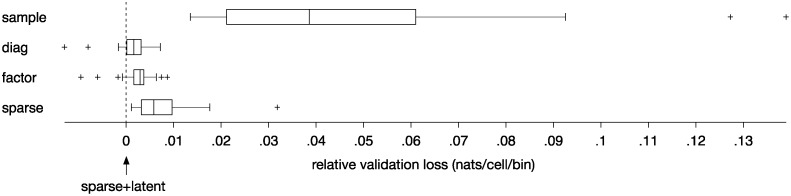
Performance of estimator *C*_sparse+latent_ expressed as validation loss (eq. 10) relative to the other estimators: *C*_sample_, *C*_diag_, *C*_factor_, and *C*_sparse_. Covariance estimators *C*_sample_, *C*_diag_, *C*_factor_, and *C*_sparse_ produced consistently greater validation losses than *C*_sparse+latent_ (*p* < 0.01 in each comparison, Wilcoxon signed rank test, *n* = 27 sites in 14 mice). The box plots indicate the 25^*th*^, 50^*th*^, and 75^*th*^ percentiles with the whiskers extending to the minimum and maximum values after excluding the outliers marked with ‘+’.

To test this hypothesis, we computed the validation loss of estimators *C*_sample_, *C*_diag_, *C*_factor_, *C*_sparse_, and *C*_sparse+latent_ in *n* = 27 imaged sites in 14 mice. The hyperparameters of each estimator were optimized by nested cross-validation (See S1 Fig. and Methods). Indeed, the sparse+latent estimator outperformed the other estimators (Fig. 4). The respective median differences of the validation loss were 0.039, 0.0016, 0.0029, and 0.0059 nats/cell/bin, significantly greater than zero (*p* < 0.01 in each comparison, Wilcoxon signed rank test).

### Structure of *C*_sparse+latent_ estimates

**Fig 5:**
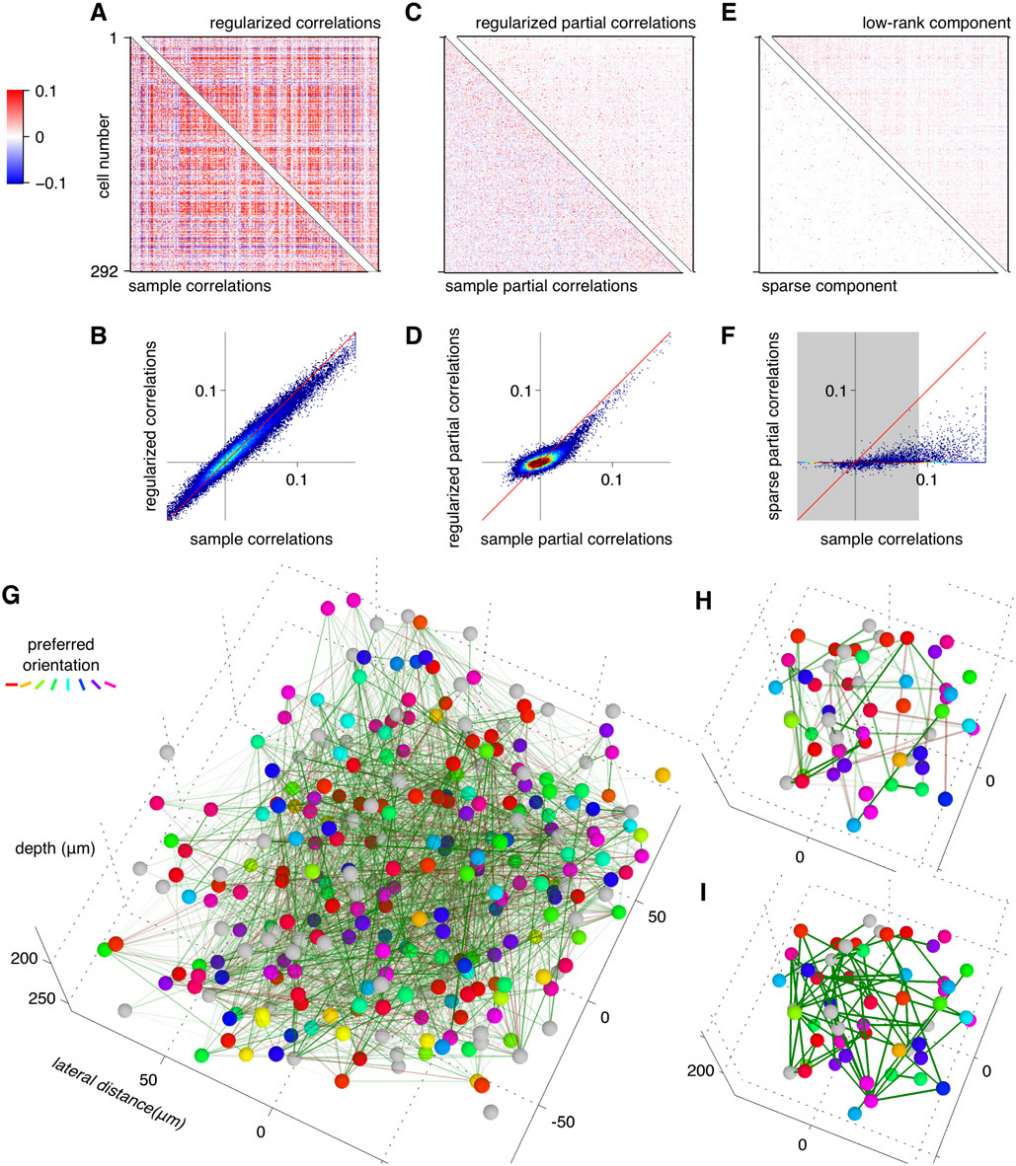
Structure revealed by *C*_sparse+latent_. **A, B**. The regularized estimate *C*_sparse+latent_ closely approximates the sample correlation matrix *C*_sample_. **C, D**. The partial correlation matrices from the two estimates differ substantially. **E.** The partial correlation matrix of the regularized estimate is decomposed into a sparse component with 92.8% off-diagonal zeros (bottom-left) and low-rank component of rank 72 (top-right). **F**. The sparse component of the regularized partial correlation matrix had little resemblance to the sample correlations: The gray region indicates the range of correlations containing 92.8% of cells pairs, equal to the fraction of zeros in the sparse partial correlation matrix. Correlation coefficients outside this interval formed the network of greatest correlations. This network differed from the sparse component of the *C*_sparse+latent_: Only 27.7% of the highest correlations coefficients outside the gray regions coincided with interactions inferred by *C*_sparse+latent_. **G.** A graphical depiction of the positive (green) and negative (magenta) sparse partial correlations as edges between observed neurons. The line weight indicates the magnitude of the partial correlation. **H.** A subset of neurons from the center of the cluster shown in **G** showing the sparse partial correlations. **I**. The same subset of neurons with edges indicating sample correlations thresholded to match the sparsity of the sparse partial correlation. These edges correspond to the sample correlation coefficients outside the gray region in panel F.

**Fig 6:**
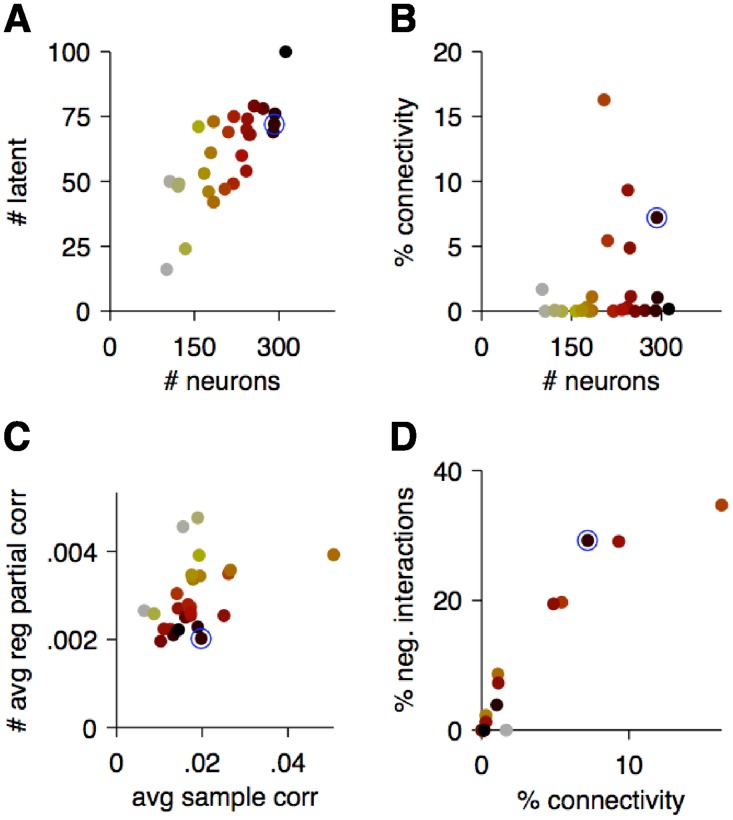
Properties of *C*_sparse+latent_ estimates from all imaged sites. Each point represents an imaged site with its color indicating the population size as shown in panels A and B. The example site from Figs. 3 and 5 is circled in blue. **A.** The number of inferred latent units *vs*. population size. **B.** The connectivity of the sparse component of partial correlations as a function of population size. **C.** The average sample correlations *vs*. the average partial correlations (Eq. 4) of the *C*_sparse+latent_ estimate. **D.** The percentage of negative interactions vs. connectivity in the *C*_sparse+latent_ estimates.

We examined the composition of the *C*_sparse+latent_ estimates for each imaged site (Fig. 5 and Fig. 6). Although the regularized estimates were similar to the sample correlation matrix (Fig. 5 A and B), the corresponding partial correlation matrices differed substantially (Fig. 5 C and D). The estimates separated two sources of correlations: a network of linear interactions expressed by the sparse component of the inverse and latent units expressed by the low-rank components of the inverse (Fig. 5 E). The sparse partial correlations revealed a network that differed substantially from the network composed of the greatest coefficients in the sample correlation matrix (Fig. 5 F, G, H, and I).

In the example site (Fig. 5), the sparse component had 92.8% sparsity (or conversely, 7.2% connectivity: connectivity = 1−sparsity) with average node degree of 20.9 (Fig. 5 G). The average node degree, *i.e.* the average number of interactions linking each neuron, is related to connectivity as degree = connectivity⋅(*p*−1), where *p* is the number of neurons. The low-rank component had rank 72, denoting 72 inferred latent units. The number of latent units increased with population size (Fig. 6 A) but the connectivity was highly variable (Fig. 6 B): Several sites, despite their large population sizes, were driven by latent units and had few pairwise interactions. This variability may be explained by differences in brain states and recording quality and warrants further investigation.

The average partial correlations calculated from these estimates according to Eq. 4 at all 27 sites were about 5 times lower than the average sample correlations (Fig. 6 C). This suggests that correlations between neurons build up from multiple chains of smaller interactions. Furthermore, the average partial correlations were less variable (*p* = 0.002 Brown-Forsythe test): the coefficient of variation of the average sample correlations across sites was 0.45 whereas that of the average partial correlations was 0.29.

While the sample correlations were mostly positive, the sparse component of the partial correlations (‘interactions’) had a high fraction (28.7% in the example site) of negative values (Fig. 5 F). The fraction of negative interactions increased with the inferred connectivity (Fig. 6 D), suggesting that negative interactions can be inferred only after a sufficient density of positive interactions has been uncovered.

Thresholded sample correlations have been used in several studies to infer pairwise interactions [26, 62–64]. We therefore compared the interactions in the sparse component of *C*_sparse+latent_ to those obtained from the sample correlations thresholded to the same level of connectivity. The networks revealed by the two methods differed substantially. In the example site with 7.2% connectivity in *C*_sparse+latent_, only 27.7% of the connections coincided with the above-threshold sample correlations (Fig. 5 F, H, and I). In particular, most of the inferred negative interactions corresponded to low sample correlations (Fig. 5 F) where high correlations are expected given the rest of the correlation matrix.

### Relationship of *C*_sparse+latent_ to orientation tuning and physical distances

**Fig 7:**
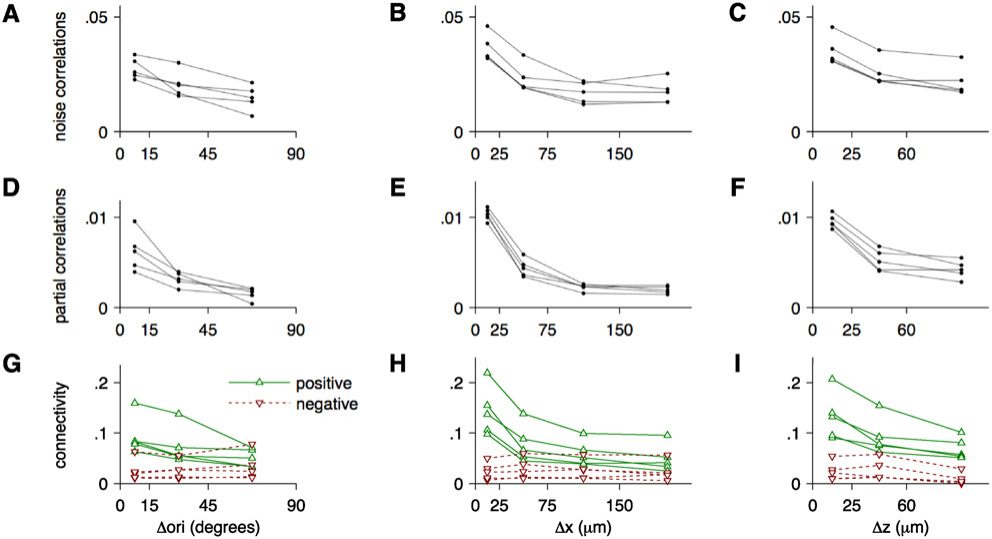
Dependence of sample correlations, regularized partial correlations, and connectivity inferred by *C*_sparse+latent_ on the differences in preferred orientations, Δori, and physical distances: horizontal Δ*x* and depth Δ*z*. Five sites with highest connectivity (see Fig. 6 B) were selected for this analysis. **A**–**C.** Mean sample correlations in relation to Δori, Δ*x* and Δ*z*, respectively. For Δ*x* averages, only horizontally aligned cell pairs with Δ*z* < 30 *μm* were considered. Similarly, for Δ*z* averages, only vertically aligned cell pairs with Δ*x* < 30 *μm* were considered. **D**–**F.** Mean partial correlations regularized by the *C*_sparse+latent_ estimator binned the same way as the sample correlations above. The partial correlations exhibit stronger dependence on Δori, Δ*x*, and Δ*z* than sample correlations. **G**–**I.** Positive connectivity (green) and negative connectivity (red) inferred by the *C*_sparse+latent_ estimator. Positive and negative connectivities refer to the fractions of the positive and negative partial correlations computed from the sparse component *S* of *C*_sparse+latent_. Positive connectivity decreases with Δori, Δ*x*, and Δ*z*. Negative connectivity does not decrease with Δori, Δ*x* within the examined range, and with Δ*z* for small values of Δ*z* < 60 *μm*.

We then examined how the structure of the *C*_sparse+latent_ estimates related to the differences in orientation preference and to the physical distances separating pairs of cells (Fig. 7). Five sites with highest pairwise connectivities were included in the analysis. Partial correlations were computed using Eq. 4 based on the regularized estimate, including both the sparse and the latent component. Connectivity was computed as the fraction of pairs of cells connected by non-zero elements (interactions) in the sparse component of the estimate, segregated into positive and negative connectivities.

First, we analyzed how correlations and connectivity depended on the differences in preferred orientations (Δori) of pairs of significantly (*α* = 0.05) tuned cells. The partial correlations decayed more rapidly with Δori than did sample correlations (Fig. 7 A and D. *p* < 10^−9^ in each of the five sites, two-sample *t*-test of the difference of the linear regression coefficients in normalized data). Positive connectivity decreased with Δori (*p* < 0.005 in each of the five sites, *t*-test on the logistic regression coefficient) whereas negative connectivity did not decrease (Fig. 7 G): The slope in the logistic model of connectivity with respect to Δori was significantly higher for positive than for negative interactions (*p* < 0.04 in each of the five sites, two-sample *t*-test of the difference of the logistic regression coefficient).

Second, we compared how correlations and connectivity depended on the physical distance separating pairs of cells. We distinguished between the lateral distance, Δ*x*, in the plane parallel to the pia, and the vertical distance, Δ*z*, orthogonal to the pia. When considering the dependence on Δ*x*, the analysis was limited to cell pairs located at the same depth with Δ*z* < 30 *μ*m; conversely, when considering the dependence on Δ*z*, only vertically aligned cell pairs with Δ*x* < 30 *μ*m were included. Again, the partial correlations decayed more rapidly both laterally and vertically than sample correlations (Fig. 7 B, C, E, F. *p* < 10^−6^ in each of the five sites, for both lateral and vertical distances, two-sample *t*-test of the difference of the linear regression coefficients in normalized data). Positive connectivity decayed with distance (*p* < 10^−6^ in each of the five sites for positive interactions, *t*-test on the logistic regression coefficient in normalized data) (Fig. 7 E, H, I), so that cells separated laterally by less than 25 *μ*m were 3.2 times more likely to be connected than cells separated laterally by more than 150 *μ*m. Although the positive connectivity appeared to decay faster with vertical than with lateral distance, the differences in slopes of the respective logistic regression models were not significant with available data. The negative connectivity decayed slower with distance (Fig. 7 H and I): The slope in the respective logistic models with respect to the lateral distance was significantly higher for positive than for negative connectivities (*p* < 0.05 in each of the five sites, two-sample *t*-test of the difference of the logistic regression coefficients).

## Discussion

### Functional connectivity as a network of pairwise interactions

Functional connectivity is often represented as a graph of pairwise interactions. The goal of many studies of functional connectivity has been to estimate anatomical connectivity from observed multineuronal spiking activity. For example, characteristic peaks and troughs in the pairwise cross-correlograms of recorded spike trains contain statistical signatures of monosynaptic connections and shared synaptic inputs [12, 14, 34, 35, 65]. Such signatures are ambiguous as they can arise from network effects other than direct synaptic connections [66]. With simultaneous recordings from more neurons, ambiguities can be resolved by inferring the conditional dependencies between pairs of neurons. Direct causal interactions between neurons produce statistical dependency between them even after conditioning on the state of the remainder of the network and external input. Therefore, conditional independence shown statistically can signify the absence of a direct causal influence.

Conditional dependencies can be inferred by fitting a probabilistic model of the joint population activity. For example, generalized linear models (GLMs) have been constructed to include biophysically plausible synaptic integration, membrane kinetics, and individual neurons’ stimulus drive [67]. Maximum entropy models constrained by observed pairwise correlations are among other models with pairwise coupling between cells [68–72]. Assuming that the population response follows a multivariate normal distribution, the conditional dependencies between pairs of neurons are expressed by the partial correlations between them. Each probabilistic model, fitted to the same data may reveal a completely different network of ‘interactions’, *i.e.*conditional dependencies between pairs of cells.

It is not yet clear which approach provides the best correspondence with anatomical connectivity. Little experimental evidence is available to answer this question. The connectivity graphs inferred by various statistical methods are commonly reported without examining their relation to anatomy. Topological properties of such graphs have been interpreted as principles of circuit organization (*e.g.* small-world organization) [62–64, 70]. However, the topological properties of functional connectivity graphs can depend on the method of inference [73]. Until a physiological interpretation of functional connectivity is established, the physiological relevance of such analyses remains in question and we did not attempt applying graph-theoretical analyses to our results.

Inference of the conditional dependencies also depends on the completeness of the recorded population: To equate conditional dependency to direct interaction between two neurons, we must record from all neurons with which the pair interacts. Unobserved portions of the circuit may manifest as conditional dependencies between observed neurons that do not directly interact. For this reason, statistical models of population activity have been most successfully applied to *in vitro* preparations of the retina or cell cultures where high-quality recordings from the complete populations were available [67]. In cortical tissue, electrode arrays record from a small fraction of cells in a given volume, limiting the validity of inference of the pairwise conditional dependencies. Perhaps for this reason, partial correlations have not, until now, been used to describe the functional connectivity in cortical populations.

Two-photon imaging of population calcium signals presents unique advantages for the estimation of functional connectivity. While the temporal resolution of calcium signals is limited by the calcium dye kinetics, fast imaging techniques combined with spike inference algorithms provide millisecond-scale temporal resolution of single action potentials [74]. However, such high temporal precision comes at the cost of lower accuracy of inferred spike rates. Better accuracy is achieved when calcium signals are analyzed on scales of tens of milliseconds [60, 75]. The major advantage of calcium imaging is its ability to characterize the spatial arrangement and types of recorded cells. Recently, advanced imaging techniques have allowed recording from nearly every cell in a volume of cortical tissue *in vivo* [59, 60] and even from entire nervous systems [76, 77]. These techniques may provide more incisive measurements of functional connectivity than electrophysiological recordings.

The low temporal resolution of calcium signals limits the use of functional connectivity methods that rely on millisecond-scale binning of signals (cross-correlograms, some GLMs, and binary maximum entropy models). Hence, most studies of functional connectivity have relied on instantaneous sample correlations [23, 26, 29, 63]. Although some investigators have interpreted such correlations as indicators of (chemical or electrical) synaptic connectivity, most used them as more general indicators of functional connectivity without relating them to underlying mechanisms.

In this study, we sought to infer pairwise functional connectivity networks in cortical microcircuits. We hypothesized that partial correlations correspond more closely to underlying mechanisms than sample correlations when recordings are sufficiently dense. Since neurons form synaptic connections mostly locally and sparsely [78], we *a priori* favored solutions with sparse partial correlations. Under the assumptions that the recorded population is sufficiently complete and that the model correctly represents the nature of interactions, the network of partial correlations can better represent the functional dependencies in the circuit than correlations.

### Functional connectivity as coactivations

Another approach to describing the functional connectivity of a circuit is to isolate individual patterns of multineuronal coactivations. Depending on the method of their extraction, coactivation patterns may be referred to as *assemblies*, *factor loadings*, *principal components*, *independent components*, *activity modes*, *eigenvectors*, or *coactivation maps* [79–84]. Coactivation patterns could be interpreted as signatures of Hebbian cell assemblies, *i.e.* groups of tightly interconnected groups of cells involved in a common computation [79, 82]. Coactivation patterns could also result from shared input from unobserved parts of the circuit, or global network fluctuations modulating the activity of the local circuit [32, 85].

Coactivation patterns and pairwise connectivity are not mutually exclusive since assemblies arise from patterns of synaptic connectivity. However, an analysis of coactivation shifts the focus from detailed interactions to collective behavior. In our study, the functional connectivity solely through modes of coactivations was represented by the factor analysis-based estimator *C*_factor_.

### Combining pairwise interactions and coactivations

In the effort to account for the joint activity patterns that are poorly explained by pairwise interactions, investigators have augmented models of pairwise interactions with additional factors such as latent variables, higher-order correlations, or global network fluctuations [32, 86–89].

In our study, we combined pairwise interactions with collective coactivations by applying the recently developed numerical techniques for the inference of the partial correlation structure in systems with latent variables [54, 55]. The resulting estimator, *C*_sparse+latent_, effectively decomposed the functional connectivity into a sparse network of pairwise interactions and coactivation mode vectors.

### Addressing ill-posedness

Inferring the conditional dependencies between variables in a probabilistic model often becomes an ill-posed problem: small variations in the data can produce large errors in the inferred network of dependencies (Fig. 5 C and D). The problem becomes worse as the number of recorded neurons increases until such models lose their statistical validity [90]. As techniques have improved to allow recording from larger neuronal populations, experimental neuroscientists have addressed this problem by extending the recording durations to keep sampling noise in check and verified that existing models are not overfitted [87]. However, ambitious projects already underway, such as the BRAIN initiative [50], aim to record from significantly larger populations. Simply increasing recording duration will be neither practical nor sufficient, and the problem must be addressed by using regularized estimators. Regularization biases the solution toward a small subspace in order to counteract the effects of sampling noise in the empirical data. However, biasing the solution to an inappropriate subspace does not allow significant estimation improvement and hinders interpretation.

Several strategies have been developed to limit the model space in order to improve the quality of the estimate. For example, Ganmor et al. [86] developed a heuristic rule to identify the most significant features that must be fitted by a maximum entropy model for improved performance in the retina. As another example of regularization, generalized linear models typically employ *L*_1_ penalty terms to constrain the solution space and to effectively reduce the dimensionality of the solution [67].

Our study demonstrates regularization schemes empirically optimized for specific types of neural data.

### Model selection

Various model selection criteria have been devised to select between families of models and the optimal subsets of variables in a given model family based on observed data. Despite its high computational demands, cross-validation is among the most popular model selection approaches due to its minimal assumptions about the data-generating process [91].

We evaluated the covariance matrix estimators using a loss function derived from the normal distribution. However, this does not limit the applicability of its conclusions to normal distributions. Other probabilistic models, fitted to the same data, could also serve as estimators of the covariance matrix. If a different model yields better estimation of the covariance matrix than the estimator proposed here, we believe that its structure should deserve consideration as the better representation of the functional connectivity.

The results of model selection must be interpreted with caution. As we demonstrated by simulation, even models with incorrect forms of dependencies can substantially improve estimates (Fig. 1). Therefore, showing that a more constrained model has better cross-validated performance than a more complex model does not necessarily support the conclusion that it reveals a better representation of dependencies in the data. This caveat is related to *Stein’s Paradox* [92]: The biasing of an estimate toward an arbitrary low-dimensional target can consistently outperform a less constrained estimate.

### Physiological interpretation and future directions

We showed that among several models a sparse network of linear interactions with several latent inputs yielded the best estimates of the noise covariance matrix for cortical microcircuits. This finding is valuable in itself: improved estimates of the noise covariance matrix for large datasets are important in order to understand the role of noise correlations in population coding [1, 6, 7, 9, 11]

Moreover, this estimation approach provides a graphical representation of the dependencies in the data that can be used to formulate and test hypotheses about the structure of connectivity in the microcircuit. Importantly, the inferred functional interactions were substantially different from the network of the highest sample correlations. For example, the *C*_sparse+latent_ estimator reveals a large number of negative interactions that were not present in the sample correlation matrix (Fig. 5 F) and may reflect inhibitory circuitry.

Distances between cells in physical space and in sensory feature space had a stronger effect on the partial correlations estimated by the *C*_sparse+latent_ estimator than on sample correlations (Fig. 7 A–F). These differences support the idea that correlations are built up from partial correlations in chains of intermediate cells positioned closer and tuned more similarly to one another, with potentially closer correspondence to anatomical connectivity. These differences may also be at least partially explained by a trivial effect of regularization: the *L*_1_ penalty applied by the estimator (Eq. 18) suppresses small partial correlations to a greater extent than large partial correlations, enhancing the apparent effect of distance and tuning. Still, the distinct positive and negative connectivity patterns (Fig. 7 G–I) may reflect geometric and graphical features of local excitatory and inhibitory networks. Indeed, the relationships between patterns of positive and negative connectivities inferred by the estimator resembled the properties of excitatory and inhibitory synaptic connectivities with respect to distance, cortical layers, and feature tuning [23, 78, 93–98]. For example, while excitatory neurons form synapses within highly specific local cliques [78], inhibitory interneurons form synapses with nearly all excitatory cells within local microcircuits [23, 96, 99]. To further investigate the link between synaptic connectivity and inferred functional connectivity, in future experiments, we will use molecular markers for various cell types with follow-up multiple whole-cell *in vitro* recordings [23, 28] to directly compare the inferred functional connectivity graphs to the underlying anatomical circuitry. Finally, the latent units inferred by the estimator can be analyzed for their physiological functions. For example, these latent units may be modulated under different brain states (e.g. slow-wave sleep, attention) and stimulus conditions (e.g. certain types of stimuli may engage feedback connections) [100, 101].

## Materials and Methods

### Ethics statement

All procedures were conducted in accordance with the ethical guidelines of the National Institutes of Health and were approved by the Baylor College of Medicine IACUC.

### Surgery and two-photon imaging

The surgical procedures and data acquisition were performed as described in [60]: C57BL/6J mice (aged p40–60) were used. For surgery, animals were initially anesthetized with isoflurane (3%). During the experiments, animals were sedated with a mixture of fentanyl (0.05 mg/kg), midazolam (5 mg/kg), and medetomidine (0.5 mg/kg), with boosts of half the initial dose every 3 hours. A craniotomy was performed over the right primary visual cortex. Membrane-permeant calcium indicator Oregon Green 488 BAPTA-1 AM (OGB-1, Invitrogen) was loaded by bolus injection. The craniotomy was sealed using a glass coverslip secured with dental cement.

Calcium imaging began 1 hour after dye injection. All imaging was performed using 3D-RAMP two-photon microscopy [60]. First, a 3D stack was acquired and cells were manually segmented. Then calcium signal were collected by sampling in the center of each cell at rates of 100 Hz or higher, depending on the number of cells.

### Visual stimulus

The visual stimulus consisted of full-field drifting gratings with 90% contrast, 10 cd/m^2^ luminance, 0.08 cycles/degree spatial frequency, and 2 cycles/s temporal frequency. Two types of stimuli were presented for each imaging site: First, directional tuning was mapped using a pseudo-random sequence of drifting gratings at sixteen directions of motion, 500 ms per direction, without blanks, with 12–30 trials for each direction of motion. Second, to measure correlations, the stimulus was modified to include only two directions of motion (in 9 datasets) or five directions (in 22 datasets) and the gratings were presented for 1 second and were separated by 1-second blanks, with 100–300 trials for each direction of motion.

### Data processing

All data were processed in MATLAB using the DataJoint data processing chain toolbox (http://datajoint.github.com).

The measured fluorescent traces were deconvolved to reconstruct the firing rates for each neuron: First, the first principal component was subtracted from the raw traces in order to reduce common mode noise related to small cardiovascular movements [60]. The resulting traces were high-pass filtered above 0.1 Hz and downsampled to 20 Hz (Fig. 3 C). Then, the firing rates were estimated using by nonnegative deconvolution [61].

Orientation tuning was computed by fitting the mean firing rates for each direction of motion *ϕ* using two-peaked von Mises tuning functions $f(\phi )=a+b\;\exp\left[\frac{1}{w}(\cos(\phi -\theta )-1)\right]+c\;\exp\left[\frac{1}{w}(\cos(\phi -\theta +\pi )-1)\right]$ where *b* ≥ *c* are the amplitudes of the two respective peaks, *w* is the tuning width, and *θ* is the preferred direction. The significance of the fit was determined by the permutation test: the labels of the direction were randomly permuted 10,000 times; the *p*-values of the fits were computed as the fraction of permutations that yielded *R* ^2^ equal to or higher than that of the original data. Cells were considered tuned with *p* < 0.05.

For covariance estimation, the analysis was limited to the period with two or five stimulus conditions and lasted between 14 and 27 minutes (mean 22 minutes). Cells that did not have substantial spiking activity (those whose variance was less than 1% of the median across the site) or whose activity was unstable (those whose variance in the least active quarter of the recording did not exceed 1% of the variance in the most active quarter) were excluded from the analysis.

### Cross-validation

To compare the performance of the estimators, we used conventional 10-fold cross-validation: Trials were randomly divided into 10 subsets with approximately equal numbers of trials of each condition in each subset. Each subset was then used as the testing sample with the rest of the data used as the training sample for estimating the covariance matrix. The average validation loss over the 10 folds was reported.

Since each of the regularized estimators had one or two hyperparameters, we used *nested cross-validation*: The outer loop evaluated the performance of the estimators with the hyperparameter values optimized by cross-validation within the inner loop. Hyperparameters were optimized by a two-phase search algorithm: random search to find a good starting point for the subsequent pattern search to find the global minimum. The inner cross-validation loop subdivided the training dataset from the outer loop to perform 10-fold cross-validation in order to evaluate each choice of the hyperparameter values. Thus the size of the training dataset within the inner loop comprised 81% of the entire recording. S1 Fig. illustrates the dependence of the validation loss on the hyperparameters of the *C*_sparse+latent_ estimator for the example site shown in Figs. 3 and 5 and the optimal value found by the pattern search algorithm.

When the validation loss was not required, only the inner loop of cross-validation was used on the entire dataset. This approach was used to compute the covariance matrix estimates and their true loss in the simulation study (Fig. 1 Rows 4 and 5) and to analyze the partial correlation structure of the *C*_sparse+latent_ estimator (Fig. 5–7).

### Covariance estimation

Within the inner loop of cross-validation, regularized covariance matrix estimation required only the sample covariance matrix *C*_sample_ of the training dataset and the hyperparameter values provided by the outer loop.

Estimator *C*_diag_ (Eq. 5) used two hyperparameters: the covariance shrinkage intensity *λ* ∈ [0, 1] and variance shrinkage intensity *α* ∈ [0, 1]. The variances (the diagonal of *C*_sample_) were shrunk linearly toward their mean value $\frac{1}{p}\;\text{Tr}(C_{\text{sample}})$:

$$D=(1-\alpha )\text{diag}(C_{\text{sample}})+\alpha \frac{1}{p}\;\text{Tr}(C_{\text{sample}})I$$ (13)

The *C*_diag_ estimate was then obtained by shrinking *C*_sample_ toward *D* according to Eq. 5.

In estimator *C*_factor_ (Eq. 6), the low-rank matrix *L* and the diagonal matrix *D* were found by solving the minimization problem

$$\left(L,D\right)=\underset{\hat{L},\hat{D}}{\text{arg}\;\text{min}}ℒ\left(\hat{L}+\hat{D},C_{\text{sample}}\right),$$ (14)

using an expectation-maximization (EM) algorithm for a specified rank of *L*. After that, the diagonal of *D* was linearly shrunk toward the its mean diagonal value similar to Eq. 13.

In estimator *C*_sparse_ (Eq. 7), the sparse precision matrix *S* was found by minimizing the *L*_1_-penalized loss with regularization parameter *λ*:

$$S=\underset{\hat{S}≻0}{\text{arg}\;\text{min}}L\;\left({\hat{S}}^{-1},C_{\text{sample}}\right)+\lambda \|\hat{S}{\|}_{1}$$ (15)

where $\hat{S}≻0$ denotes the constraint that $\hat{S}$ be a positive-definite matrix and $\|\hat{S}{\|}_{1}$ is the element-wise *L*_1_ norm of the matrix $\hat{S}$. This problem formulation is known as *graphical lasso* [102, 103]. To solve this minimization problem, we adapted the alternative-direction method of multipliers (ADMM) [55]. Unlike *C*_diag_ and *C*_factor_, this estimator does not include linear shrinkage: the selection of the sparsity level provides sufficient flexibility to fine-tune the regularization level.

Estimator *C*_sparse+latent_ (Eq. 8) estimates a larger sparse precision matrix S* of the joint distribution of the *p* observed neurons and *d* latent units.

$$S^{*}=\left(\begin{array}{cc} S & S_{12}\\ S_{12}^{T} & S_{22} \end{array}\right),$$ (16)

where the *p* × *p* partition *S* corresponds to the visible units. Then the covariance matrix of the observed population is

$$C_{\text{sparse}+\text{latent}}={\left(S-S_{12}S_{22}^{-1}S_{12}^{T}\right)}^{-1}$$ (17)

The rank of the *p*×*p* matrix $L=S_{12}S_{22}^{-1}S_{12}^{T}$ matches the number of the latent units in the joint distribution. Rather than finding *S*_12_ and *S*_22_ separately, *L* can be estimated as a low-rank positive semidefinite matrix. To simultaneously optimize the sparse component *S* and the low-rank component *L*, we adapted the loss function with an *L*_1_ penalty on *S* and another penalty on the trace of *L* [54, 55]:

$$\left(S,L)=\underset{\hat{S},\hat{L}}{\text{arg}\;\text{min}}\;L{\left(\hat{S}-\hat{L}\right)}^{-1},C_{\text{sample}}+\alpha \|\hat{S}{\|}_{1}+\beta \;\text{Tr}(\hat{L}\right)$$ (18)

The trace of a symmetric semidefinite matrix equals the sum of the absolute values of its eigenvalues, *i.e.* its *nuclear norm*; penalty on Tr(*L*) favors solutions with few non-zero eigenvalues or, equivalently, low-rank solutions while keeping the convexity of the overall optimization problem [104, 105]. This allows using convex optimization algorithm such as ADMM to be applied with great computational efficiency [55].

The partial correlation matrix (Eq. 4) computed from *C*_sparse+latent_ includes interactions between the visible and latent units and was used in Fig. 5 C and D and Fig. 6 C, and Fig. 7 D–F). The partial correlation matrix computed from *S* alone expresses strengths of pairwise interactions

$$P_{\text{sparse}}=-{\left(\text{diag}(S)\right)}^{-\frac{1}{2}}S{\left(\text{diag}(S)\right)}^{-\frac{1}{2}}$$ (19)

and were used in Fig. 5 F, G, H.

The MATLAB code for these computations is available online at http://github.com/atlab/cov-est.

### Cross-validation with conditioned variances

Special attention was given to estimating the variances. All evaluations and optimization in this study were defined with respect to the covariance matrices. However, neuroscientists often estimate a common correlation matrix across multiple stimulus conditions when the variances of responses are conditioned on the stimulus [106, 107]. In this study, we too conditioned the variances on the stimulus but estimated a single correlation matrix across all conditions. Here we describe the computation of the validation loss (Eq. 10) when the variances were allowed to vary with the stimulus condition.

Let *T*_*c*_ and $T_{c}^{\prime }$ denote the sets of time bin indices for the training and testing samples, respectively, limited to condition *c*.

Similar to Eq. 2, the training and testing sample covariance matrices for condition *c* are

$$C_{c,\text{sample}}=\frac{1}{n_{c}}\sum_{t\in T_{c}}\left(x(t)-{\bar{x}}_{c}\right){\left(x(t)-{\bar{x}}_{c}\right)}^{T}$$ (20)

and

$$C_{c,\text{sample}}^{\prime }=\frac{1}{n_{c}^{\prime }}\sum_{t\in T_{c}^{\prime }}\left(x(t)-{\bar{x}}_{c}\right){\left(x(t)-{\bar{x}}_{c}\right)}^{T}$$ (21)

Here *n*_*c*_ and $n_{c}^{\prime }$ denote the sizes of *T*_*c*_ and $T_{c}^{\prime }$, respectively.

Note that ${\bar{x}}_{c}=\frac{1}{n_{c}}\sum_{t\in T_{c}}x(t)$ is estimated from the training sample but used in both estimates, making $C_{c,\text{sample}}^{\prime }$ an unbiased estimate of the true covariance matrix, Σ. As such, $C_{c,\text{sample}}^{\prime }$ can be used for validation.

The common correlation matrix *R*_sample_ is estimated by averaging the condition-specific correlations:

$$R_{\text{sample}}=\frac{1}{n}\sum_{c}n_{c}\left(V_{c,\text{sample}}^{-\frac{1}{2}}C_{c,\text{sample}}V_{c,\text{sample}}^{-\frac{1}{2}}\right)=\frac{1}{n}\sum_{c}\sum_{t\in T_{c}}z(t)z{\left(t\right)}^{T},$$ (22)

where $n=\sum_{c}n_{c}$ and *V*_*c*, sample_ = diag(*C*_*c*, sample_) is the diagonal matrix containing the sample variances. Then *R*_sample_ is simply the covariance matrix of the *z*-score signal $z(t)=V_{c,\text{sample}}^{-\frac{1}{2}}\left(x(t)-{\bar{x}}_{c}\right)$ of the training sample.

For consistency with prior work, we applied regularization to covariance matrices rather than to correlation matrices. The common covariance matrix was estimated by scaling *R*_sample_ by the average variances across conditions $V_{\text{sample}}=\frac{1}{n}\sum_{c}n_{c}V_{c,\text{sample}}$:

$$C_{\text{sample}}=V_{\text{sample}}^{\frac{1}{2}}R_{\text{sample}}V_{\text{sample}}^{\frac{1}{2}}$$ (23)

Note that *C*_sample_ differs from the sample covariance matrix computed without conditioning the variances on *c* and this computation helps avoid any biases that would be introduced by ignoring changes in variance.

The covariance matrix estimators *C*_diag_, *C*_factor_, *C*_sparse_ or *C*_sparse+latent_ convert *C*_sample_ into its regularized counterpart denoted here as *C*_reg_.

To evaluate the estimators, we regularized the conditioned variances by linear shrinkage toward their mean value across all conditions. This was done by scaling *C*_reg_ by the conditioned variance adjustment matrix $Q_{c}=\delta I+(1-\delta )V_{\text{sample}}^{-1}V_{c,\text{sample}}$ to produce the conditioned regularized covariance matrix estimate:

$$C_{c,\text{reg}}=Q_{c}^{\frac{1}{2}}C_{\text{reg}}Q_{c}^{\frac{1}{2}}$$ (24)

The variance regularization parameter *δ* ∈ [0, 1] was optimized in the inner loop of cross-validation along with the other hyperparameters.

The overall validation loss is obtained by averaging the validation losses across all conditions:

$$\frac{1}{\sum_{c}n_{c}^{\prime }}\sum_{c}n_{c}^{\prime }L\left(C_{c,\text{reg}},C_{c,\text{sample}}^{\prime }\right)$$ (25)

With negative normal log-likelihood as the validation loss (Eq. 10) and the unbiased validation covariance matrix *C*_*c*, sample_, the loss function in Eq. 25 is an unbiased estimate of the true loss. Hence, it was used for evaluations reported in Fig. 4.

### Simulation

For simulation, ground truth covariance matrices were produced by taking 150 independent samples from an artificial population of 50 independent, identically normally distributed units. The covariance matrices were then subjected to the respective regularizations to produce the ground truth matrices for the simulation studies (Fig. 1 Row 2). Samples were then drawn from multivariate normal distributions models with the respective true covariance matrices to be estimated by each of the estimators. For Ising models, the negative inverse of the true covariance matrix was used as the matrix of coupling coefficients and the sampling was performed by the Metropolis-Hastings algorithm.

## Supporting Information

S1 Fig Optimization of hyperparameters of the *C*_sparse+latent_ estimator. **A.** Validation loss (Eq. 25) for the example site in Fig. 3 and 5 as a function of the hyperparameters *α* and *β* of the *C*_sparse+latent_ estimator (Eq. 8 and Eq. 18). In all panels, the red cross marks the optimal value found by the pattern search algorithm described in Methods. **B.** The connectivity (1 − sparsity) of the sparse component *S* as a function of *α* and *β* for the example site. **C.** The number of latent units, *i.e.* the rank of the low-rank component *L*, as a function of hyperparameters *α* and *β*. **D.** The loss function as a function of the connectivity and the number of latent units.
(EPS)
